# Management and Outcomes of Postoperative Airway Obstruction in Patients After Tissue‐Augmentation Palatoplasty

**DOI:** 10.1002/ohn.1319

**Published:** 2025-05-29

**Authors:** Pooja D. Reddy, Soukaina Eljamri, Amber D. Shaffer, Matthew Ford, Rachel Whelan, Allison Tobey, Noel Jabbour

**Affiliations:** ^1^ University of Pittsburgh School of Medicine Pittsburgh Pennsylvania USA; ^2^ UPMC Children's Hospital of Pittsburgh Pittsburgh Pennsylvania USA

**Keywords:** airway obstruction, cleft palate, pediatrics, polysomnography, tissue‐augmentation palatoplasty

## Abstract

**Objective:**

To characterize postoperative airway obstruction and evaluate management strategies in pediatric patients with cleft palate following tissue‐augmentation palatoplasty (TAP).

**Study Design:**

Retrospective case series.

**Setting:**

Single academic center.

**Methods:**

Patients with obstruction within 1 year of primary TAP between 2017 and 2023 were identified. Fisher's exact, Wilcoxon rank‐sum, and Spearman rank correlation were used to investigate the relationship between TAP type, obstruction severity, interventions, and polysomnography (PSG) findings (Obstructive Apnea‐Hypopnea Index [OAHI], total Apnea‐Hypopnea Index [AHI]) and disposition details.

**Results:**

Of the 129 patients who underwent primary TAP, 25 patients developed obstructive symptoms (19.4%); 52% female, 32% syndromic. In total, 17 underwent surgical intervention for obstruction (68.0%): revision palatoplasty/flap revision (8/25, 35%), tonsillectomy and partial cephalic adenoidectomy (10/25, 40%), and partial cephalic adenoidectomy only (5/25, 20%). In total, 11 were medically managed and 3 were observed without intervention. In nine patients with paired PSGs, there was no difference in pre‐TAP and post‐TAP AHI or OAHI. Patients who underwent surgical revision had worse pre‐TAP AHI compared to those who did not undergo surgical revision (mean ± SD: 15.7 ± 5.9 vs 6.2 ± 4.1, *P* = .01).

**Conclusion:**

TAP is a newer surgical technique used to address tissue deficiency in cleft palate repair. Most patients who experienced postoperative obstruction following TAP ultimately required surgical intervention, though preoperative AHI may help identify those at higher risk for obstruction. Future studies are necessary to evaluate the efficacy of earlier interventions and elucidate factors impacting obstruction risk and symptom resolution.

Orofacial clefts are the most common human craniofacial congenital anomaly, significantly impacting speech mechanics due to palatal defects and abnormal orientation of the levator veli palatini (LVP) muscle.^1^, ^2^ This leads to velopharyngeal dysfunction (VPD) and reduced speech quality. Palatoplasty is performed to close the palatal defect, with the Furlow double‐opposing Z‐plasty or straight‐line techniques with intravelar veloplasty being the current standards. However, traditional palatoplasty may not fully address the tissue deficiency inherent to orofacial clefts, leading to complications such as dehiscence, scarring, oronasal fistula formation, and VPD.^3^

Techniques like pharyngoplasty aim to improve VPD by narrowing the nasopharyngeal port, but carry risks such as mouth breathing and obstructive sleep apnea (OSA).^4^, ^5^ Newer surgical techniques include tissue‐augmentation palatoplasty (TAP) with buccinator myomucosal flaps (BMFs) and/or buccal fat pad transfers (BFPTs), which lengthen nasal and oral lining, reduce scarring, reposition the LVP sling, and fill dead space with vascularized tissue.^6^ Despite these theoretical advantages, the postoperative outcomes of TAP remain relatively unexplored. Anecdotal evidence suggests that TAP may be associated with higher rates of postoperative airway obstruction due to the increased tissue volume, which can present as snoring or apneic episodes. Management of TAP‐related obstructive complications remains undefined. We aim to describe the characteristics and current management of postoperative airway obstruction following TAP in pediatric patients with cleft palate.

## Methods

This study was approved by the University of Pittsburgh Institutional Review Board (STUDY19100247). A retrospective review was conducted of patients who underwent primary TAP at a tertiary pediatric center between 2017 and 2023. Patients with postoperative airway obstruction symptoms within 1 year of surgery were included. Exclusion criteria included no subjective or objective postoperative obstruction, no workup for mild symptoms, symptoms appearing >1 year post‐TAP, and objective preoperative OSA diagnosis. Covariates included Veau classification, type of TAP performed (buccal fat and/or myomucosal flaps), gender, age, weight, gestational age, and presence of syndromes or other medical diagnoses.

Outcomes of interest included obstruction symptoms, timing of obstruction, interventions, and symptom resolution. Subjective obstruction symptoms were identified per parent report in clinic or emergency department (ED) visit notes. Objective evidence of obstruction was determined using polysomnography (PSG) metrics, including Obstructive Apnea‐Hypopnea Index (OAHI), total Apnea‐Hypopnea Index (AHI), and O_2_ nadir (collected both preoperatively and postoperatively when available). OAHI and AHI reflect obstructive and total apnea and hypopnea events per hour of total sleep time.^7^ An OSA diagnosis was made with AHI > 1/hour or OAHI > 1/hour. These metrics were also used to compare the impact of the intervention type on obstruction. Nasolaryngoscopy findings were also noted. Interventions (ie, observation, medical, surgical, and intubation) were detailed, with intervention type, timing of the intervention, and evidence of subsequent symptom resolution. Disposition details included total hospital days during admission for TAP, length of any postoperative pediatric intensive care unit (PICU) stay, and number of ED visits within 1 year post‐TAP for obstruction.

Categorical data were summarized as n (%). Continuous data were summarized as mean (standard deviation, SD) when normally distributed (Shapiro‐Wilk *P* > .05) and as median (range) when not. Patient characteristics, PSG findings, symptom resolution, length of stay, and ED visits for obstruction were compared between the following groups: (1) those who underwent TAP with bilateral buccal fat and BMFs versus other techniques, (2) those who underwent revision palatoplasty versus those who did not, (3) those who underwent revision division and inset of myomucosal flaps versus those who did not, (4) those who had surgical intervention for obstruction versus those managed medically or with observation, and (5) those with resolution of obstructive symptoms versus those without. Associations between PSG findings and patient characteristics, symptom resolution, length of stay, or ED visits for obstruction were also evaluated. For factors significantly associated with post‐TAP PSG parameters, the initial univariate analysis was followed by multivariable regression to account for preoperative parameters. Statistical analyses were performed in Stata/SE 16.1 (StataCorp) using Fisher's exact test, Wilcoxon rank‐sum, *t* tests, and Spearman rank with *α* = .05.

## Results

### Demographics

In total, 129 patients were reviewed, and 25 met the inclusion criteria (19%), with 52% being female (13/25) and 32% (8/25) being syndromic (Table 1). All syndromic patients had a diagnosis of Robin sequence, and 5/8 patients had surgically corrected micrognathia with mandible osteotomy and distractor placement before TAP. Two patients (8%) had Veau I clefts, 18 patients (72%) had Veau II clefts, 4 patients (16%) had Veau III clefts, and 1 patient (4%) had a Veau IV cleft. Three patients had a history of prematurity (gestational age <28 weeks), 13 (52%) had congenital heart disease, and 15 (60%) underwent airway‐related surgeries including lip and nose repair, mandibular distraction, supraglottoplasty, or bilateral turbinoplasty. The mean age and weight at the time of palatoplasty were 13 months (range: 12‐23 months) and 9 kg (SD 1.5 kg), respectively. One patient (1/25; 4%) underwent tissue augmentation with bilateral myomucosal flaps only, 4 (16%) underwent TAP with bilateral fat only, 6 (24%) underwent TAP with bilateral fat and unilateral myomucosal flaps, and 14 (56%) underwent TAP with bilateral fat and bilateral myomucosal flaps.

**Table 1:** Demographics and Type of Tissue‐Augmentation Palatoplasty (TAP)

Demographics	n (%)
Female	13/25 (52.0%)
Cleft lip and palate	5/25 (20.0%)
Premature	3/25 (12.0%)
Congenital heart disease	13/25 (52.0%)
Robin sequence	8/25 (32.0%)

	Mean (SD)
Weight at procedure, kg	9.0 (1.5)

	Median (range)
Age, mo	13 (12‐23)

Type of TAP	n (%)
Bilateral myomucosal flaps only	1/25 (4.0%)
Bilateral fat only	4/25 (16.0%)
Bilateral fat and unilateral myomucosal flaps	6/25 (24.0%)
Bilateral fat and bilateral myomucosal flaps	14/25 (56.0%)

### Obstructive Symptoms

Of the 25 included patients, 13 (52%) displayed symptoms of snoring, mouth breathing, and/or restlessness only, whereas the remaining 12 (48%) experienced these symptoms along with more severe symptoms of gasping, pausing, and/or witnessed apnea. The average time from TAP to obstructive symptom onset was 113 days (SD 89.2; range: 1‐321 days). In total, 16 patients underwent nasal endoscopy post‐TAP, 13 of which displayed evidence of obstruction. Obstruction was defined as >50% adenoid obstruction, >3+ tonsils, evidence of tongue base collapse, or explicitly noted narrow airways or excessive tissue obstruction.

### Interventions

**Figure 1:**
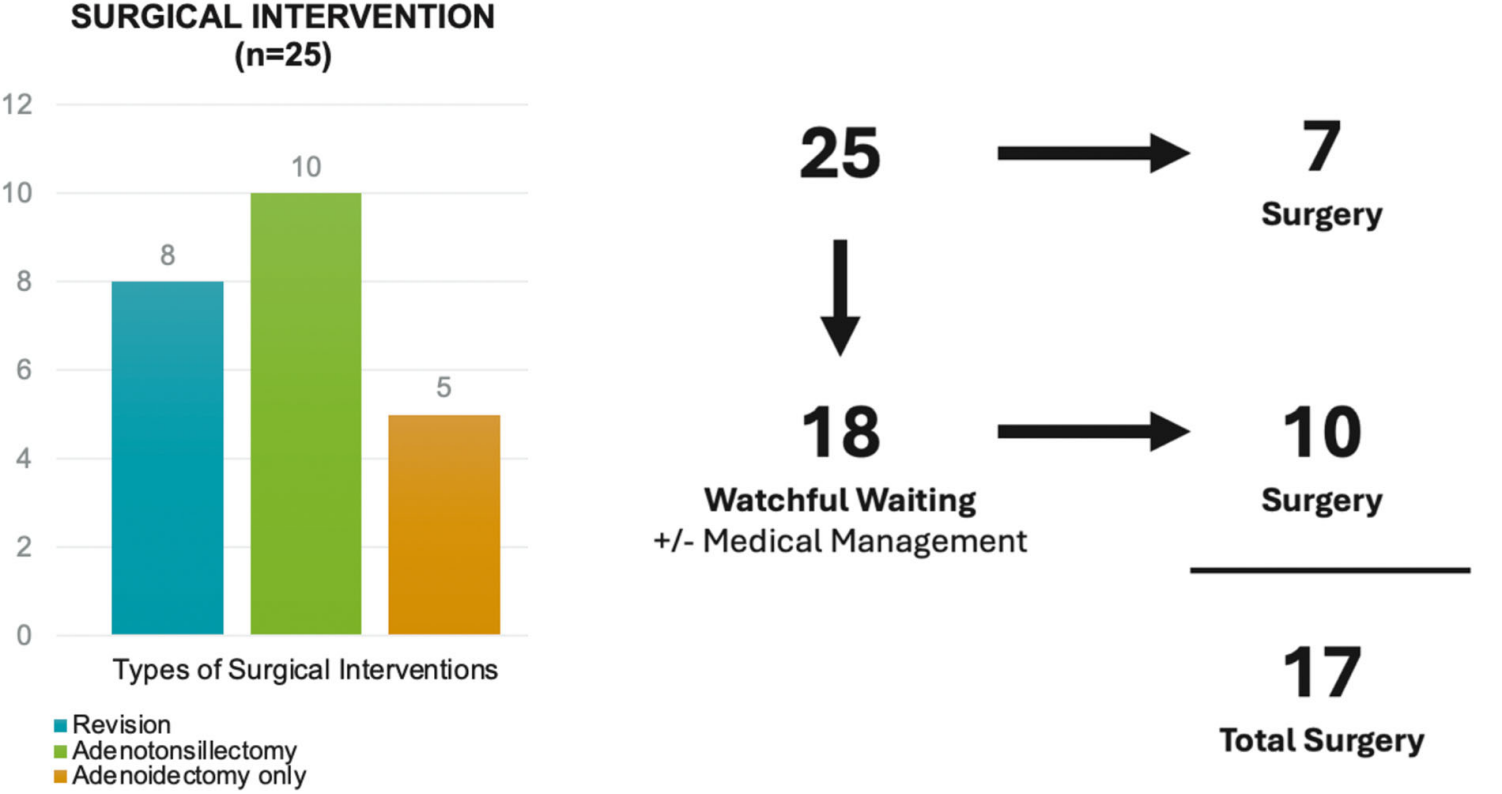
Types of surgical interventions performed in pediatric patients following TAP and a schematic illustrating the total patients who underwent surgical intervention.

In this cohort, the initial intervention was observation in 36% of patients (9/25), medical intervention in 36% of patients (9/25), and surgical intervention in 28% (7/25). Four of those who were initially observed and six who underwent initial medical management subsequently progressed to surgical management. Eleven patients (44%) were managed medically with or without subsequent surgical intervention, with four patients (16%) being managed medically only, and three (12%) were observed without any intervention. Of the three patients who underwent observation only, two presented with snoring only without apnea, and the third presented with restlessness and reported witnessed apnea in addition to snoring. The decision to observe the patient was made after an uneventful postoperative sleep study that showed OAHI and AHI values of 0.7 and 2.1, respectively. Surgical intervention was completed for 17 (68%) patients. Surgical interventions included revision palatoplasty and/or division and inset of myomucosal flap pedicles (8/25, 35%), tonsillectomy and partial cephalic adenoidectomy (T&A) (10/25, 40%), and partial cephalic adenoidectomy only (5/25, 20%) (Figure 1). Among the eight patients who underwent revision, three had both revision palatoplasty with BMF division and inset, three had flap inset only, one had lysis of the left BMF pedicle, and one underwent bilateral mandibular reconstruction with inverted osteotomy and placement of mandibular distractors. One revision palatoplasty with BMF division and inset was performed to address posterior velar dehiscence and concomitant hypernasality, as well as airway obstruction. The remaining seven revisions were indicated for clinical symptoms of airway obstruction. Medical interventions included oral and nasal antihistamines, nasal corticosteroids, nasal decongestants, and oral leukotriene receptor antagonists.

### PSG Findings

**Figure 2:**
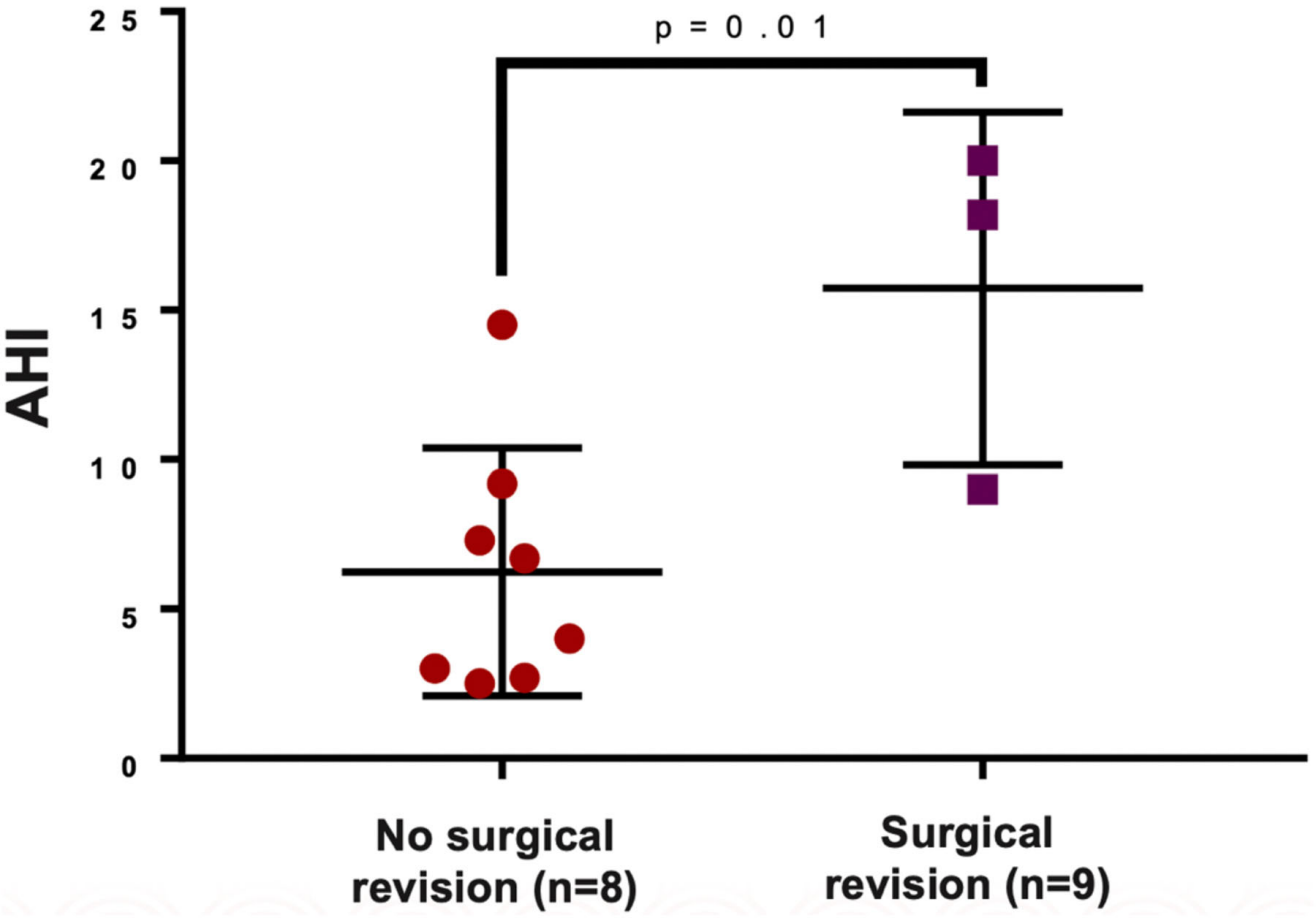
Comparison of preoperative Apnea‐Hypopnea Index (AHI) in pediatric patients who required surgical revision versus those managed without surgery.

Eleven patients underwent pre‐TAP PSGs, 22 underwent post‐TAP PSGs, and 9 underwent both. Patients who underwent surgical revision (revision palatoplasty or flap revision) had a significantly worse preoperative AHI than those who did not (mean ± SD: 15.7 ± 5.9 vs 6.2 ± 4.1, *P* = .01) (Figure 2). Among patients with no prior intervention before their first postoperative PSG, those who underwent surgical revision had a lower postoperative O_2_ nadir (median, range: 82, 78‐86 vs 88, 57‐91, *P* = .045), though this was not significant after adjusting for preoperative O_2_ nadir.

Males had significantly worse median OAHI (19.2, range 1.6‐117) compared to females (2.7, range 0.2‐22.4) (*P* = .046), and patients who underwent T&A had worse OAHI (20.8, range 2.8‐117) than those who did not (1.8, range 0.2‐68.3) (*P* = .01). Worse OAHI and O_2_ nadir were both associated with longer hospital stays (Spearman's rho = 0.584, *P* = .01, and Spearman's rho = −0.681, *P* = .003, respectively) and more ED visits (Spearman's rho = 0.525, *P* = .03).

**Figure 3:**
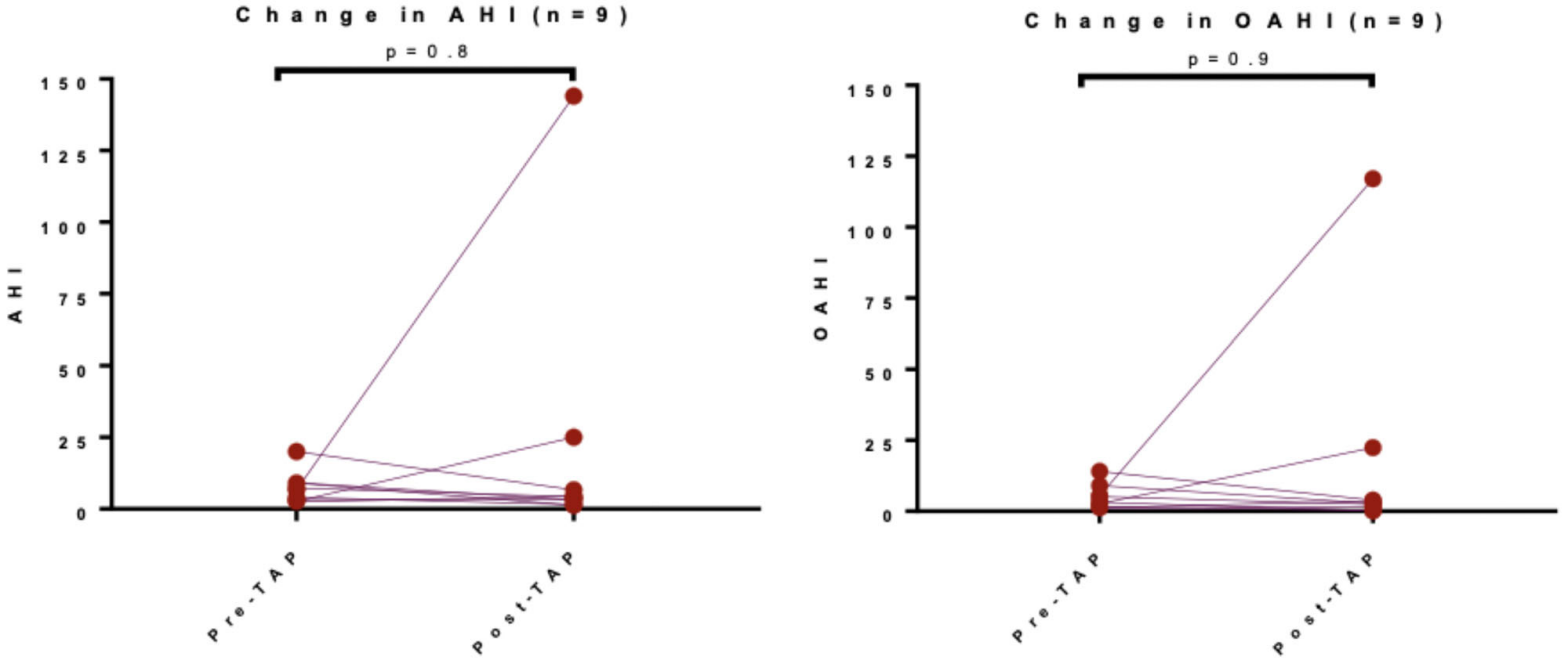
Pre‐tissue‐augmentation palatoplasty (pre‐TAP) and post‐TAP comparison of Apnea‐Hypopnea Index (AHI) and Obstructive Apnea‐Hypopnea Index (OAHI) in pediatric patients with paired polysomnography data.

In the nine patients with both pre‐TAP and post‐TAP PSGs, there was no significant difference in the magnitude of improvement in AHI or OAHI between those managed surgically and those managed medically (Figure 3). Seven of the nine had Robin sequence, with five undergoing mandible osteotomy and distractor placement correction prior to TAP. The other two of the nine patients had comorbidities, including transposition of the great vessels, severe developmental delays, dysphagia with aspiration, facial asymmetry, adrenal insufficiency, torticollis, and scoliosis.

### Outcomes of Intervention

Six patients experienced resolution of symptoms within 1 year of their initial obstruction; one patient had unknown resolution status (6/24, 25%). Resolution of symptoms within 1 year was associated with the bilateral fat with bilateral flap TAP technique, with 46.2% (6 out of 13) experiencing symptom resolution compared to 0% (0 out of 11) with other techniques (*P* = .02). Symptom resolution was also more common in those with Robin sequence compared with those without (4/7, 57.1% vs 2/17, 11.8%, *P* = .04).

### Disposition

The average total number of hospital days during the admission for primary palatoplasty was 2.92 days (range: 1‐10 days). Four patients had postoperative intensive care unit (ICU) stays, staying for an average of 4.5 days in the ICU (range: 2‐9 days). Three patients presented to the ED for obstruction. Patient 1 developed apnea 7 months after TAP with unilateral myomucosal flaps and bilateral fat and was observed overnight for desaturations. Patient 2 presented with respiratory distress and wheezing 10 weeks postoperatively and was treated with a nebulizer. Patient 3 required PICU admission 5 weeks postoperatively for upper airway obstruction and later returned 5 months postoperatively in respiratory distress, requiring intubation and hospitalization. All described ED visits had negative viral panels.

## Discussion

In this case series, we outline the management and outcomes of postoperative airway obstruction in a cohort of pediatric patients following TAP. Most patients exhibited symptoms such as snoring, mouth breathing, and restlessness, whereas a subset experienced more severe manifestations, including gasping and witnessed apnea. Initial management primarily involved observation or medical approaches, with over half eventually requiring surgical intervention. Adenotonsillectomy (T&A) was the most common procedure, alleviating OSA symptoms in more than 80% of cases, making it the preferred surgical option.^8^ However, successful outcomes with medical intervention alone in our cohort suggest that a conservative approach may suffice initially, warranting observation without intervention for mild obstructive symptoms.

Interestingly, preoperative and postoperative PSG metrics did not show significant differences, contrary to our expectation of worse OSA post‐TAP due to the additional tissue. This discrepancy may be attributed to our limited paired data; of the nine patients with both pre‐TAP and post‐TAP PSGs, seven had Robin sequence, and the other two had complex medical histories. The associations between Robin sequence and postoperative airway obstruction would suggest that these patients would be at higher risk for requiring subsequent interventions; however, this was not observed.^9^ Notably, five of the seven underwent mandibular osteotomy and distractor placement before TAP, which may have mitigated the expected impact of additional tissue on airway outcomes.

Patients who underwent preoperative T&A had worse postoperative AHI, likely due to higher baseline risk for airway obstruction, as T&A was performed in those with more severe OSA or predisposing airway anatomy. In some cases, T&A alone may not have fully resolved obstruction, and TAP may have added further airway narrowing due to increased soft tissue bulk. Additionally, early T&A may contribute to later pharyngeal collapse due to alterations in airway support structures.^10^

The addition of BMFs to the Furlow double‐opposing Z‐plasty for repair of wider cleft palates was first described in 1997.^11^ The rising popularity of the addition of BMFs is due to the significant incidence of superficial dehiscence or oronasal fistula post‐Furlow palatoplasty.^12^ Qiu et al hypothesize that the buccal fat flap primarily provides an additional vascular layer that supplies the overlying oral mucosa, thus buffering against vascular compromise and breakdown.^13^ They further hypothesize that the fat flap serves to fill the dead space between the oral and nasal mucosal Z‐plasty flaps, reducing the likelihood of subsequent vascular compromise or dehiscence. With the increased use of TAP in cleft repair, postoperative speech outcomes are being explored in the literature. In a review of 505 patients undergoing double‐opposing Z‐plasty with or without BMFs for cleft palate repair, Mann et al found no significant differences in nasal resonance scores or the need for secondary speech surgery between groups, with most patients achieving normal speech and nasal resonance postoperatively.^14^ Similarly, Anstadt et al examined tissue augmentation in revision palatoplasty for persistent velopharyngeal insufficiency in 20 patients, showing significant improvements in speech, with the Pittsburgh Weighted Speech Score (PWSS) dropping from 14.3 to 4.2 (*P* < .001).^6^ Although speech outcomes following TAP are actively being explored, airway outcomes and management of severe obstruction have been less characterized.

Based on our findings and the practice at our institution, we have outlined a multidisciplinary management framework for managing airway obstruction following TAP. Children with obstructive symptoms post‐TAP are initially evaluated by otolaryngologists with a clinical assessment to gauge the severity of obstruction, noting symptoms like snoring, mouth breathing, restlessness, gasping, pausing, and witnessed apnea. Flexible fiberoptic laryngoscopy may be performed to assess airway patency and identify the level of obstruction. PSG is also proposed based on the extent of symptoms to further characterize obstructive apneic episodes with objective metrics including AHI, OAHI, and oxygen desaturations. Interventions are determined based on the severity of symptoms. For mild symptoms, such as snoring only with minimal PSG abnormalities, observation with close monitoring is utilized. Medical management is initiated for mild to moderate symptoms, such as snoring, mouth breathing, and restlessness with or without apnea, and includes antihistamines, corticosteroids, decongestants, and leukotriene antagonists. Surgical intervention is reserved for severe symptoms, including significant apnea, gasping, pausing, or PSG values indicating more severe OSA or those with recurrent severe nasal infections secondary to obstruction. If significant hypertrophy of tonsils and/or adenoids is noted on exam causing obstructive symptoms, tonsillectomy and/or partial cephalic adenoidectomy is considered. For those with significant nasopharyngeal fullness or those not responding to medical management, revision palatoplasty or flap revision may be indicated. PSG findings guided the decision for surgery, with worse pre‐op AHI/OAHI linked to requiring surgery, longer hospital stays, and more ED visits for obstruction; therefore, more careful and frequent follow‐up may be indicated for these patients. Additionally, in those with subjective preoperative OSA symptoms, optimization was performed before surgery when possible, using medical interventions such as topical nasal steroids and montelukast. Preoperative optimization, such as mandibular distraction, may reduce the need for intervention in Robin sequence patients. After standard postoperative follow‐up, long‐term follow‐up included evaluations every 3 to 9 months based on the severity of symptoms and frequency of interventions. ED visits for severe obstruction were managed based on presenting symptoms and generally required immediate interventions such as nebulizers, antibiotics, nasal sprays, and potential intubation. This framework is based on our institution's current approach for managing postoperative obstruction in pediatric patients following TAP in a small cohort of patients and varies depending on patient and provider characteristics and preferences.

When comparing the management of TAP‐related obstruction with similar obstructive etiologies such as tonsillar and adenoid hypertrophy, a similar stepwise approach is observed. Initial clinical assessment followed by fiberoptic evaluation helps characterize obstruction patterns by otolaryngologists.^15^ Medical management with nasal corticosteroids is considered for mild symptoms (snoring, rhinorrhea, mouth breathing, and cough) and demonstrates short‐term success, with varying degrees of efficacy.^15^, ^16^ PSG remains the gold standard for diagnosing sleep‐disordered breathing in these patients and informing subsequent surgical intervention like T&A.^17^ Interestingly, preoperative PSG can aid in identifying children at a greater risk of persistent OSA following T&A, as a high preoperative AHI was associated with increased risk for residual sleep‐disordered breathing following surgery.^18^ Similarly, preoperative AHI in our study was also clinically useful for predicting the likelihood of requiring further interventions post‐TAP, as children with higher preoperative AHI scores were more likely to experience severe obstructive symptoms that necessitated surgical management. This predictive capability of preoperative PSG may warrant standardized inclusion in the evaluation and treatment planning for pediatric patients undergoing TAP.

Our study is limited by its retrospective nature and small sample size. Additionally, there were inconsistencies in the timing intervals of PSG, and not every patient had both preoperative and postoperative PSG for comparison. The scope findings and patient report outcomes were subjective and not consistently reported with variable time intervals, further limiting the study's generalizability. The average time from TAP to the onset of obstructive symptoms in our cohort was 113 days, with patients presenting up to 321 days postoperatively. Although obstruction is often expected to manifest earlier, delayed presentation may result from a combination of progressive tissue changes, scarring, and barriers to healthcare access. Potential changes in fat volume over time could also contribute to gradual airway narrowing.^19^ We included syndromic patients to ensure this important cohort was represented; however, given the additional risk factors, future studies could isolate this group to reduce variability. Future studies could also investigate the timing of interventions and proper follow‐up to address postoperative obstruction. Standardized postoperative follow‐up protocols may mitigate variable patient‐reported outcomes. Additionally, studies may consider prospective analysis of post‐TAP outcomes and the utility of preoperative PSG in predicting postoperative risks.

## Conclusion

This case series characterizes postoperative airway obstruction following primary TAP in pediatric patients with orofacial clefts. We found a spectrum of obstructive symptoms post‐TAP, from mild snoring to severe apneic episodes, necessitating varied management approaches. Notably, higher preoperative AHI and OAHI correlated with more severe postoperative outcomes, highlighting the importance of preoperative PSG. Our treatment framework highlights the effectiveness of a conservative, stepwise approach by a multidisciplinary team of providers. Physicians should be vigilant to anticipate and to assess for postoperative airway obstruction for this relatively new set of procedures.

## Author Contributions

**Pooja D. Reddy**, conceptualization, data curation, investigation, methodology, writing–original draft preparation, review and editing; **Soukaina Eljamri**, conceptualization, data curation, writing–review and editing; **Amber D. Shaffer**, conceptualization, formal analysis, methodology, writing–review and editing; **Matthew Ford**, data curation, writing–review and editing; **Rachel Whelan**, supervision, writing–review and editing; **Allison Tobey**, conceptualization, methodology, project administration, supervision, writing–review and editing; **Noel Jabbour**, conceptualization, methodology, project administration, supervision, writing–review and editing.

## Disclosures

### Competing interests

The authors have no conflicts of interest to declare.

### Funding source

The authors have no financial disclosures.
